# Synchronous parotid and nasopharyngeal Warthin's tumor: case report and literature review^☆^

**DOI:** 10.1016/j.bjorl.2017.05.009

**Published:** 2017-06-27

**Authors:** Tom Ben-Dov, Evgeny Edelstein, Ben I. Nageris, Firas Kassem

**Affiliations:** a Meir Medical Center, Department of Otolaryngology Head and Neck Surgery, Kfar Saba, Israel; b Tel Aviv University, Sackler Faculty of Medicine, Tel Aviv, Israel; c Meir Medical Center, Department of Pathology, Kfar Saba, Israel

## Introduction

Warthin's tumor, also known as papillary cystadenoma lymphomatosum, was first described in 1895 as a variant of a lateral cervical cyst consisting of bilayered oncocytic and basal epithelium forming cystic structures, papillae and glands that are accompanied by a dense lymphoid stroma.^1^ The eponymous Aldred Scott Warthin published two case reports in 1929 presenting with the same morphology described by Hildebrand^1^ 34 years earlier.^2^

Among parotid gland tumors, Warthin's tumor is relatively common (∼10%), second only to pleomorphic adenoma (∼80%). Warthin's tumor shows male predominance and commonly appears in the sixth to seventh decades, rarely before age 40. As it is related to smoking, current trends show a decline in male incidence paralleled by an increase in female incidence, probably due to the decline in smoking among men and a reverse trend among women.^3^ Warthin's tumor is found almost exclusively in the parotid gland, and in 7–10% of cases, is present bilaterally. Here we present a case of synchronous Warthin's tumors in the parotid and nasopharynx and provide an updated literature review.

## Case report

**Figure 1:**
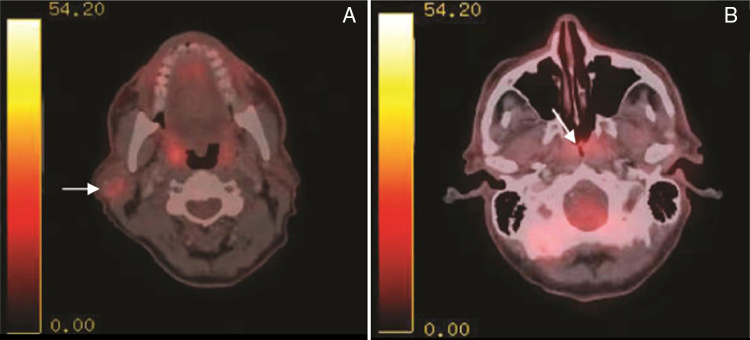
PET-CT with intravenous injection of f-18 Fludeoxyglucose (FDG). (A) White arrow – right sided parotid mass enhancement. (B) White arrow nasopharyngeal enhancement.

**Figure 2:**
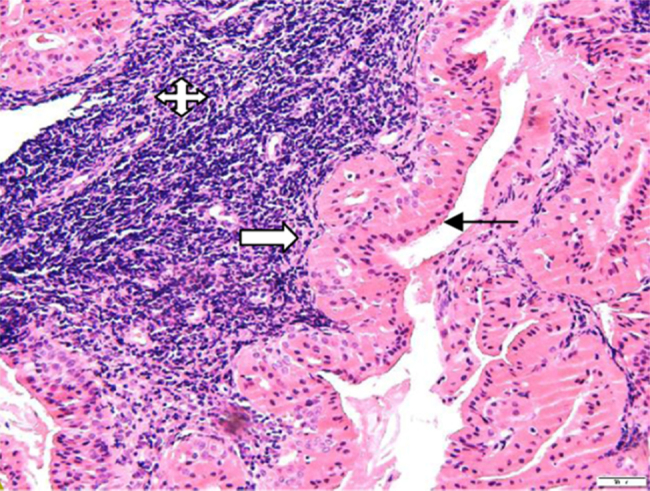
Histologic examination revealed papillary and cystic lesions comprised of epithelial and lymphoid cells (H&E stain, 200×). The epithelial component shows a double layer of granular eosinophilic/oncocytic cells: luminal non-ciliated columnar cells with nuclei aligned toward the lumen (black arrow) and basal round or polygonal basal cells having vesicular nuclei (white arrow). The lymphoid component was composed of mature small lymphocytes (quad arrow) (Image 7883, H&E stain, 200×).

**Figure 3:**
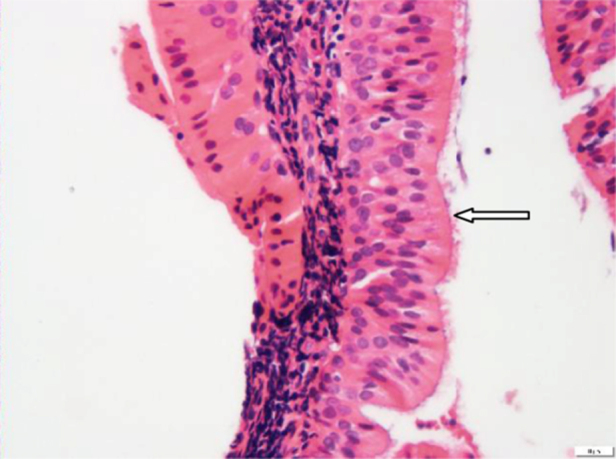
Nasopharyngeal lining columnar ciliated epithelium (white arrow) is not involved (H&E stain, 400×).

**Figure 4:**
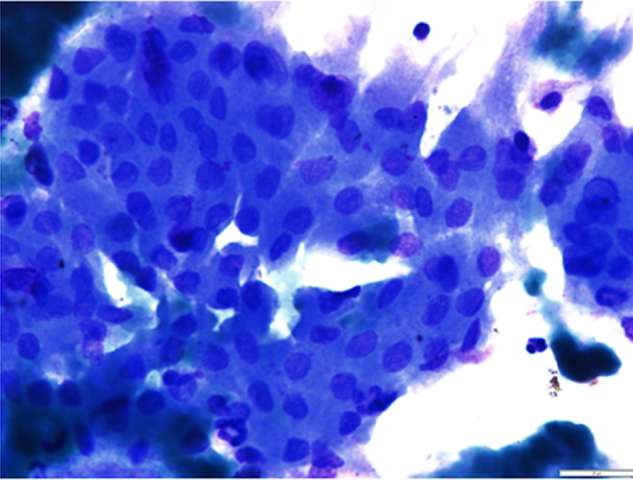
Fine needle aspiration from parotid gland lesion shows clustered oncocytic epithelium, with honeycomb arrangement, surrounded by a few lymphocytes. The epithelial cells have uniform round nuclei, and moderate finely granular cytoplasm (Papanicolaou stain, 400×).

A 65-year-old retired electrician was referred to the Otolaryngology Department in our University-affiliated medical center. He had recently undergone Positron Emission Computed Tomography (PET-CT) to reevaluate the extent of his advanced squamous cell lung cancer. His medical history was consistent with heavy smoking, hypercholesterolemia and deep vein thrombosis. Upon receipt of results, two unexpected lesions – one at the level of the mandibular angle (Fig. 1A) and the other at the back of the nasal cavity (Fig. 1B) – were noticed. The patient underwent nasopharyngeal biopsy (Figure 2, Figure 3) and fine needle aspiration (FNA) of the parotid gland (Fig. 4). Two small fragments of tan-brown soft tissue, 0.5 cm and 0.4 cm in diameter, were sent for pathological examination.

Both “incidentilomas” were found to be Warthin's tumor, a benign salivary gland tumor not directly related to the patient's background morbidity. Considering the patient's medical history, he was not suitable for a surgical procedure at the time of diagnosis and remained as such until his death two years later.

We conducted a systematic literature search of PubMed/Medline and Clinicalkey databases through October 2016 using the search terms “Warthin's, Nasopharyngeal Warthin's and Synchronous Warthin's Tumor. Fourteen articles met the inclusion criteria, among which seven documented cases of synchronous parotid and nasopharyngeal Warthin's tumor were identified. Including the current case report, we reviewed eight patients.

Nasopharyngeal Warthin's tumor is very rare and only a few cases have been published to date. Synchronous nasopharyngeal and parotid Warthin's tumors are even more exceptional, with just seven cases previously documented. In our review, we identified male predominance (7:1) and overall mean age of 62.5 years (Table 1). Including the current report, five of the reviewed patients had history of smoking.^4^, ^5^, ^6^, ^7^ There was no documentation regarding the rest, except one was known to have COPD.^8^ Two patients had otological complaints as a presenting symptom.^8^, ^9^ Two patients had bilateral parotid glands and nasopharyngeal area diagnosed with Warthin's tumor.^5^, ^9^ Four patients presented with unilateral mass at the mandibular angle.^4^, ^6^, ^7^, ^10^ Due to lack of documentation, we were not able to identify common systemic factors.

**Table 1:** Synchronous parotid gland and nasopharyngeal Warthin's tumor reported in the literature.

N°	Gender	Age	Medical history	Smoking	Symptoms	Reference
1	M	77	ACS, HTN, DM	Yes	Painful mass left side of neck, weight loss, dysphonia	Yeh et al.^4^
2	M	55	NA	Yes	Bilateral submandibular mass	Güçlü^5^
3	M	64	IHD, HTN	Yes	Mandibular angle mass	Hilton et al.^6^
4	M	63	DM	Yes	Mandibular angle mass	Yanez-Barraza et al.^7^
5	M	71	HTN, gout, COPD, CRF	N/A	Audiologic	Low and Ng^8^
6	M	52	NA	N/A	SOM, bilateral mass in parotid region	Ory and Eran^9^
7	F	53	NA	N/A	Right upper neck mass	Pelucchi et al.^10^
8	M	65	SCC lung, dyslipidemia	Yes	None	Current case

M, male; F, female; ACS, acute coronary syndrome; HTN, hypertension; DM, diabetes mellitus; IHD, ischemic heart disease; COPD, chronic obstructive pulmonary disease; CRF, chronic renal failure; N/A, not available; SOM, serous otitis media; SCC, squamous cell carcinoma.

## Discussion

In this review, we describe a patient incidentally diagnosed with Warthin's tumor while undergoing an evaluation for pulmonary neoplasm. Although it is possible that the appearance of two simultaneous Warthin's tumors and the pulmonary malignancy was coincidental, it is interesting to consider a possible association.

Various medical disciplines, including maxillofacial surgeons, perform parotidectomy. A physical examination by an otolaryngologist includes fiberoptic evaluation of the nasopharyngeal space. The current report describes eight patients with Warthin's tumor synchronously present in the parotid gland and in the nasopharynx. Without the nasopharyngeal evaluation by an otolaryngologist, the second tumor might have been overlooked. Malignant transformation of Warthin's tumor is very rare (less than 1% of cases) and none of the reviewed cases reported this sequela. Risk factors for Warthin's tumor are known; however, no single risk factor has been identified for multifocal disease.

## Conclusion

The diagnosis of synchronous parotid and nasopharyngeal Warthin's tumor in our report was established based on PET-CT imaging our patient had undergone for a systemic disease. In the current report it is likely that without this imaging the diagnosis would have been overlooked. Parotid gland masses are easily noticed due the superficial location of the gland. Having noted this however, nasopharyngeal masses are characteristically more insidious in nature. Since parotidectomy is performed by various surgical disciplines, we suggest that patients undergo fiberoptic nasopharyngeal examination by an otolaryngologist before surgery.

## Conflicts of interest

The authors declare no conflicts of interest.
